# Enhanced Ocular Delivery of Epalrestat Using Nanostructured Lipid Carrier Laden Soft Contact Lens

**DOI:** 10.3390/pharmaceutics17121515

**Published:** 2025-11-24

**Authors:** Ketan Ranch, Yashkumar Patel, Esha Acharya, Paras Gupta, Anil Kumar Singh, Sudarshan Singh

**Affiliations:** 1 Department of Pharmaceutics and Pharmaceutical Technology, L. M. College of Pharmacy, Ahmedabad 380009, Gujarat, India; ketan.ranch@lmcp.ac.in (K.R.); acharyaesha0@gmail.com (E.A.); 2 Research Scholar, PhD Section (Pharmacy), Gujarat Technological University, Ahmedabad 382424, Gujarat, India; 3 Department of Pharmacognosy, United Institute of Pharmacy, Prayagraj 211010, Uttar Pradesh, India; parasgupta.uip@gmail.com; 4 Department of Pharmaceutices, United Institute of Pharmacy, Prayagraj 211010, Uttar Pradesh, India; singhanil2682@gmail.com; 5 Office of Research Administration, Chiang Mai University, Chiang Mai 50200, Thailand; 6 Faculty of Pharmacy, Chiang Mai University, Chiang Mai 50200, Thailand

**Keywords:** epalrestat, nanostructured lipid carrier, contact lens, ocular drug delivery, diabetic retinopathy, sustained release

## Abstract

**Background/Objectives**: Epalrestat (EPL), an aldose reductase inhibitor, exhibits poor aqueous solubility and limited ocular bioavailability, which significantly restricts its therapeutic efficacy in the treatment of diabetic retinopathy. To overcome these limitations, a novel nanostructured lipid carrier (NLCs)-laden contact lens system was developed to achieve sustained and enhanced ocular delivery of EPL. **Methods**: In this study EPL-loaded NLCs were prepared using Compritol^®^ 888 ATO (solid lipid), Labrafac™ WL 1349 (liquid lipid), and Solutol^®^ HS 15 (surfactant) using high-speed homogenization method. The formulations were statistically optimized using a D-optimal mixture design, considering globule size (Y_1_), swelling index (Y_2_), and drug release at 6 h (Y_3_) as key responses. The optimized NLCs were incorporated into contact lenses via the soaking technique and evaluated for physicochemical properties, drug content, in vitro release, ex vivo corneal permeability, and in vivo ocular tolerance. **Results**: The optimized NLCs formulation showed a globule size of 41.85 ± 2.14 nm, zeta potential of −20.3 ± 1.8 mV, and entrapment efficiency of 93.32 ± 1.27%, indicating excellent physical stability with high drug encapsulation. The swelling index of the optimized NLCs-laden contact lens was 140.69 ± 4.32%, and the optical transmittance was 80.54 ± 1.12%, confirming adequate hydration and transparency for ocular use. The drug content was 96.32 ± 0.84%, ensuring uniform distribution throughout the hydrogel matrix. In vitro release studies demonstrated a sustained drug release of 98.12 ± 2.08% over 24 h, whereas ex vivo corneal permeation indicated significantly higher permeation (97.26 ± 1.95% at 6 h) compared with the control contact lens (38.14 ± 2.41% at 5 h). The in vivo Draize test confirmed that both blank and drug-loaded contact lenses were non-irritating and biocompatible. **Conclusions**: Thus, the optimized EPL NLCs-laden contact lens demonstrated enhanced corneal permeation, prolonged drug retention, and excellent ocular safety, offering a promising advancement in the management of diabetic retinopathy by improving bioavailability, reducing dosing frequency, and enhancing therapeutic efficacy.

## 1. Introduction

Diabetic retinopathy (DR) is one of the most serious complications of diabetes mellitus and remains a major cause of preventable blindness worldwide [1,2,3]. Small retinal microaneurysms and vascular leakage are the first signs of the disease, which develops silently. Later stages involve neovascularization, vitreous hemorrhage, or retinal detachment [4,5]. Further contributing to vision loss is diabetic macular oedema, which is brought on by fluid accumulation in the macula and disruption of the blood–retinal barrier [6,7]. Clinical results have improved with modern therapies like anti-VEGF injections, corticosteroid implants, and laser photocoagulation. However, they limit long term patient compliance because they are intrusive, expensive, and need to be administered repeatedly [5,8]. These drawbacks emphasize the necessity of non-invasive delivery methods that can offer long-term eye treatment. Chronic hyperglycemia-induced oxidative stress, the production of advanced glycation end products, the activation of protein kinase-C, and stimulation of the polyol pathway are all part of the multifactorial pathogenesis of diabetic retinopathy [9,10,11]. The polyol pathway is one of the most significant of these. Aldose reductase in this pathway uses NADPH to convert glucose to sorbitol. Sorbitol builds up in hyperglycemic environments, causing oxidative damage, angiogenesis, and osmotic imbalance, all of which contribute to retinal damage [12,13,14]. The only aldose reductase inhibitor currently approved for clinical use is epalrestat (EPL), a carboxylic acid derivative that is sold in Japan to treat diabetic neuropathy [15]. Significant inhibition is produced when its carboxyl group attaches to the anion pocket of the enzyme and hydrophobic interactions maintain the binding [14,16,17].

EPL has significant formulation issues despite its potential. This medication, which is classified as a Class II drug by the Biopharmaceutics Classification System (BCS), has low ocular tissue distribution and limited oral bioavailability due to its poor aqueous solubility and dissolution [18]. Barriers to topical eye drops include poor corneal permeability and fast tear turnover, which result in less than 5% of the medication reaching intraocular tissues [19]. Advanced delivery techniques that enhance solubility, ocular retention, and targeted release are necessary to overcome these constraints. Numerous methods have been studied to deal with these problems. Alvarez-Rivera et al. reported silicone hydrogel lenses functionalized with reversible binding groups, which improved corneal accumulation and allowed EPL release for up to a week [20]. Kattar et al. developed cationic niosomes that achieved high encapsulation efficiency and enhanced corneal-scleral permeation [21], while oleo-gels containing natural gelators showed promising ex vivo and vivo results [22]. In a separate study, Alvi et al. created cyclodextrin inclusion complexes embedded in chitosan nanoparticles that enhanced preclinical models’ safety, pharmacokinetics, and dissolution [23]. All these studies show how new platforms like vesicular carriers, contact lenses, and nanoparticle complexes can help get past the delivery obstacles of EPL.

Recently, nanostructured lipid carriers (NLCs) have become popular as adaptable delivery systems for hydrophobic medications such as EPL. NLCs create a matrix with structural flaws by mixing liquid and solid lipids, which improves drug loading, solubility, and stability [19]. Their lipid composition ensures safety and sustained release, while their nanoscale size facilitates corneal penetration [24]. Concurrently, therapeutic soft contact lenses are becoming more well-known as multifunctional tools that can both correct vision and act as drug reservoirs [25]. Lenses significantly enhance ocular distribution by avoiding precorneal clearance and delivering medication straight to the corneal surface [26,27]. Soft contact lenses and NLCs work together to deliver EPL in a potent way. While the lens ensures extended retention and non-invasive administration, NLCs address problems with solubility and encapsulation. For chronic conditions like DR, where consistent, patient-friendly treatment is essential, this combination is extremely valuable.

Therefore, the development and characterization of EPL-loaded NLCs-laden soft contact lenses are the primary aims of this study. Enhancing solubility, encapsulation effectiveness, corneal penetration, and sustained release while preserving important lens characteristics like comfort and transparency is the objective. By integrating NLCs with contact lens technology, this work seeks to provide an effective, non-invasive, and patient-compliant strategy for the management of diabetic retinopathy.

## 2. Materials and Methods

EPL (Mw: 319.42 g mol^−1^; >98%) was provided as a gift sample from Symed Labs Ltd., Hyderabad, India. Caprylic/capric triglycerides (Labrafac™ WL 1349; 0.95 g cm^−3^), caprylocaproyl polyoxyl-8 glycerides (Labrasol^®^; 1.05 g cm^−3^), polyglycol mono- and di-esters of 12-hydroxystearic acid 30% PEG (Solutol^®^ HS15; 1.10 g cm^−3^), and glyceryl behenate (Compritol^®^ 888 ATO; 1.03 g cm^−3^) were generously supplied by Gattefosse India Pvt. Ltd., Mumbai, India. Octanoic acid (caprylic acid; 0.91 g cm^−3^) was purchased from Subhash Chemical Industries, Mumbai, India. Octadec-9-enoic acid (oleic acid; 0.88 g cm^−3^) was obtained from S.D. Fine Chemicals, Mumbai, India. Isopropyl myristate (0.85 g cm^−3^), polysorbate 80 (Tween^®^ 80; 1.06 g cm^−3^), polysorbate 60 (Tween^®^ 60; 1.08 g cm^−3^), sorbitan monolaurate (Span^®^ 20; 1.03 g cm^−3^), sorbitan monooleate (Span^®^ 80; 0.99 g cm^−3^), stearic acid, palmitic acid, lauric acid, glyceryl monostearate, and poloxamer 188 (Lutrol^®^ F68; 1.14 g cm^−3^) were procured from Sigma-Aldrich Chemicals Pvt. Ltd., Bangalore, India. Hydroxyethyl methacrylate (HEMA; 130.14 g mol^−1^), methacrylic acid (MAA; 86.09 g mol^−1^; 1.02 g cm^−3^), and ethylene glycol dimethacrylate (EGDMA; 198.22 g mol^−1^) were also purchased from Sigma-Aldrich Chemicals Pvt. Ltd., Bangalore, India. Diphenyl (2,4,6-trimethylbenzoyl) phosphine oxide (Darocur^®^ TPO) was supplied by Tokyo Chemical Industry (TCI) Pvt. Ltd., Nihonbashi-kodemmacho, Chuo-ku, Tokyo. Polyvinyl alcohol (PVA; Mw: 85,000–124,000 g mol^−1^, 87–89% hydrolyzed) was obtained from Loba Chemie Pvt. Ltd., Mumbai, India. All other chemicals and solvents used in this study were of analytical grade.

### 2.1. Solubility Study of EPL in Solid Lipid, Liquid Lipid and Surfactant

The solubility of EPL in various excipients was determined to screen suitable components for NLCs formulation. An excess quantity of EPL was added to 2.0 mL of each selected lipid or surfactant in screwcap glass vials. The solid lipids investigated were stearic acid, glyceryl monostearate, palmitic acid, lauric acid, and Compritol^®^ 888 ATO. Liquid lipids included Labrafac™ WL 1349, caprylic acid, oleic acid, isopropyl myristate, and Labrasol^®^. Surfactants assessed were polysorbate 80 (Tween^®^ 80), polysorbate 60 (Tween^®^ 60), Solutol^®^ HS15, poloxamer 188, sorbitan monolaurate (Span^®^ 20), and sorbitan monooleate (Span^®^ 80). The mixtures were vortexed for 5 min to enhance initial drug dispersion and then incubated in an orbital shaker (100 rpm, 37 ± 0.5 °C) for 24 h to reach equilibrium solubilization. Following equilibration, samples were transferred to Eppendorf tubes and centrifuged at 7000 rpm for 10 min using a refrigerated centrifuge (Sorvall Legend X1R, Thermo Scientific, Waltham, MA, USA) to separate undissolved residues. The clear supernatant was carefully withdrawn, diluted with methanol, and analyzed using a UV–Visible spectrophotometer (Shimadzu UV-1800, Japan) at 389 nm to determine the solubility of EPL in each excipient.

### 2.2. Preparation of EPL Loaded Nanostructured Lipid Carrier

**Figure 1:**
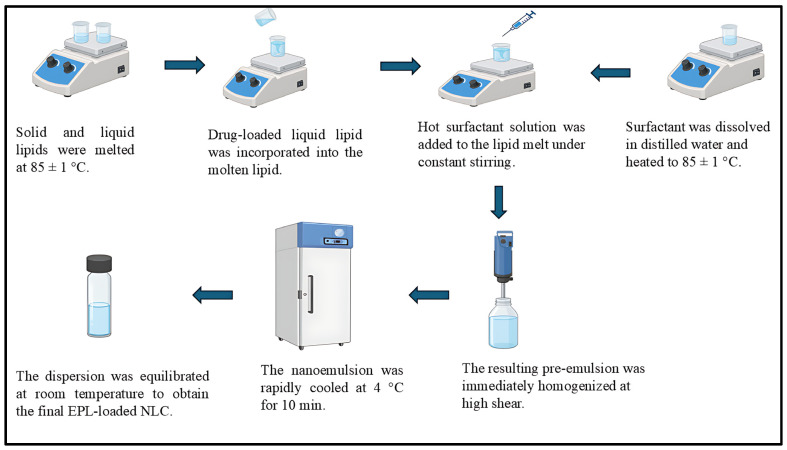
Preparation of EPL loaded nanostructured lipid carrier.

EPL loaded NLCs were fabricated by the high shear homogenization technique as showed in Figure 1. Briefly, Compritol^®^ 888 ATO (solid lipid) and Labrafac™ WL1349 (liquid lipid) were weighed and heated in a water bath at 85 ± 1 °C until a clear, homogeneous lipid melt was obtained. EPL was dissolved in the liquid lipid and subsequently incorporated into the molten lipid phase under continuous stirring at 85 °C. In parallel, the aqueous phase was prepared by dissolving Solutol^®^ HS15 in distilled water and heating to the same temperature (85 ± 1 °C) to prevent premature lipid solidification. The hot aqueous surfactant solution was added dropwise to the lipid melt under constant stirring to form a coarse pre-emulsion. The pre-emulsion was immediately subjected to high shear homogenization (Ultra-Turrax^®^, IKA, Staufen, Germany) at 15,000 rpm for 10 min to reduce droplet size and obtain a stable nanoemulsion. The resulting nanoemulsion was rapidly cooled in an ice bath at 4 °C for 10 min to induce lipid recrystallization, thereby solidifying the lipid matrix and forming NLCs. The dispersion was then equilibrated at room temperature to yield the final EPL loaded NLCs formulation.

### 2.3. Optimization of NLC Using Optimal Mixture Design

A D-optimal mixture design strategy was employed to optimize the composition of EPL loaded NLCs. Three formulation variables solid lipid (Compritol^®^ 888 ATO), liquid lipid (Labrafac™ WL1349), and surfactant (Solutol^®^ HS15) were considered as independent factors, each evaluated at two different levels, selected on the basis of preliminary solubility studies. The design aimed to assess the effect of these excipients on three critical quality attributes (CQAs) mean globule size (Y_1_, nm), swelling index (%) of the NLCs-laden contact lenses (Y_2_), and cumulative drug release (%) at 6 h from the contact lenses prepared by the soaking method (Y_3_). The coded levels of independent and dependent variables are summarized in Table 1. Experimental runs were generated according to the mixture design matrix (Table 2) using Design-Expert^®^ software (version 13, Stat-Ease Inc., Minneapolis, MN, USA). Analysis of variance (ANOVA) was performed to determine the statistical significance of model terms, identify the optimal composition, and evaluate model adequacy. An overlay plot was used to define the ideal area, and contour and 3D surface plots were used to further visualize the design space. The optimized formulation obtained through this approach was prepared and subjected to comprehensive evaluation to confirm the predicted responses.

### 2.4. Characterization of Batches of EPL Loaded Nanostructured Lipid Carrier

#### 2.4.1. Globule Size, Polydispersity Index, and Zeta Potential Analysis

The mean globule size, polydispersity index (PDI), and zeta potential (ZP) of the EPL-loaded NLCs formulations were determined by dynamic light scattering (DLS) using a Zetasizer Nano ZS90 (Malvern Instruments Ltd., Worcestershire, UK). Prior to analysis, freshly prepared samples were diluted with double-distilled water to avoid multiple scattering effects and sonicated for 5 min in an ultrasonic cleaner (LMU6, LABMAN, Mumbai, India) to ensure homogenous dispersion and minimize aggregation. Measurements were performed at a controlled temperature of 25 ± 0.1 °C with a fixed scattering angle of 90°. Each sample was analyzed in triplicate, and results were reported as mean ± standard deviation (SD).

#### 2.4.2. Entrapment Efficiency

The entrapment efficiency (EE %) of EPL-loaded NLCs was determined by an indirect method [28]. Briefly, the NLCs dispersion was centrifuged at 10,000 rpm for 10 min at 25 ± 1 °C (Remi Equipment Pvt. Ltd., Mumbai, India) to separate the free drug from the lipid matrix. The supernatant was collected, appropriately diluted with methanol, and analyzed using UV–Visible spectrophotometry (Shimadzu UV-1800, Kyoto, Japan) at 389 nm to quantify the concentration of unentrapped drug (*W_free_*) with reference to straight line equation obtained from standard curve of native EPL. The entrapment efficiency was calculated using the following equation:

$$EE\;\%=\frac{W_{total}-W_{free}}{W_{total}}\times 100$$

where *W_total_* is the total amount of drug used in the formulation and *W_free_* represents the unentrapped drug present in the supernatant. All measurements were performed in triplicate, and the results were reported as mean ± SD.

### 2.5. Fabrication of Contact Lenses

Contact lenses were fabricated using a free-radical polymerization method. The monomer mixture consisted of hydroxyethyl methacrylate (HEMA, 70% *v*/*v*) as the base monomer, ethylene glycol dimethacrylate (EGDMA, 1.5% *v*/*v*) as the crosslinker, methacrylic acid (MAA, 0.5% *v*/*v*) as the co-monomer, Darocur^®^ (0.5% *w*/*v*) as the photo-initiator, and distilled water (28% *v*/*v*) to obtain a homogeneous solution. The cast-moulding technique was employed, wherein the monomer solution was poured into pre-cleaned polypropylene moulds. Polymerization was initiated by exposing the mould assembly to ultraviolet (UV) light at 366 nm for 10 min, resulting in the formation of contact lens. To preserve hydration and sterility, the contact lenses were carefully taken out of the moulds once they had been cured and placed in sterile vials filled with simulated tear fluid (STF).

### 2.6. Preparation of EPL-Loaded NLC-Laden Contact Lenses

The soaking method was used to incorporate EPL into contact lenses. To facilitate drug absorption, each blank contact lens was submerged in 10 mL of NLCs dispersions and allowed to sit at room temperature for 24 h. The contact lenses were carefully taken out after incubation, rinsed with simulated tear fluid to remove any surface-adhered particles, and coded as HE1–HE22, which correspond to the corresponding NLCs formulations listed in Table 2. Contact lenses were soaked in a drug solution containing 10 mg of EPL dissolved in 10 mL of simulated tear fluid under the same conditions to create a control batch for comparison. These control contact lenses were labeled as HL1. The control group enabled direct comparison of drug entrapment efficiency and release behavior between simple soaking in drug solution and NLCs mediated loading.

### 2.7. Characterization of EPL Loaded NLC-Laden Contact Lens

#### 2.7.1. Swelling Study

The swelling behaviour of the fabricated contact lenses was evaluated to assess their water absorption capacity. The dry weight (W_d_) of each contact lens was recorded after removal from the moulds and complete drying at room temperature. The dried contact lenses were then immersed in 3 mL of STF at room temperature. At predetermined time intervals, the contact lenses were removed, gently blotted with filter paper to eliminate excess surface fluid, and reweighed until a constant mass was obtained, corresponding to the swollen weight (W_s_). The swelling percentage (%) was calculated using the following equation

$$Swelling(\%)=\frac{W_{s}-W_{d}}{Wd}\times 100$$

#### 2.7.2. Optical Clarity Study

The optical transmittance of the fabricated contact lenses was evaluated using a UV–Visible spectrophotometer (Shimadzu Corporation, Kyoto, Japan). The contact lenses were fixed inside a quartz cuvette, and the percentage transmittance was recorded at 630 nm against distilled water as the reference. To make sure reproducibility, the study was carried out in triplicate (*n* = 3) for each prepared formulation.

#### 2.7.3. Determination of Drug Content in Contact Lenses

UV-Vis spectroscopy (Shimadzu, Japan) was employed to measure the drug content in EPL-loaded NLCs-laden contact lenses. First, to eliminate moisture, the contact lenses were removed from their packaging solution and dried in a hot air oven at 40 °C for one hour. After drying, the contact lenses were broken into smaller pieces and immersed in 5 mL of methanol to ensure complete drug extraction. They were then shaken at 100 rpm for 24 h at room temperature using an orbital shaker. Finally, the extracts were analyzed at a wavelength of 389 nm, and their drug content was compared to a methanol blank.

### 2.8. In Vitro Release

In vitro release of EPL loaded NLCs-laden contact lenses was evaluated in STF. Each contact lens was immersed in 3 mL of STF and incubated in an orbital shaker at 34 ± 2 °C with constant agitation at 100 rpm to mimic physiological tear turnover. At predetermined time intervals (1, 2, 3, 4, 5, 6,7, 12 and 24 h), 3 mL aliquots of the release medium were withdrawn and replaced with an equal volume of fresh STF to maintain sink conditions. The drug content in the collected samples was quantified using UV–Visible spectrophotometry at 400 nm against blank STF. The cumulative percentage of drug released was calculated, and release profiles were plotted as a function of time to evaluate the sustained-release behaviour of the NLCs-laden contact lenses.

### 2.9. Ocular Irritancy Test

The ocular irritancy test was conducted following the approved animal experimentation protocol (LMCP/IAEC/2024/5/149) sanctioned by the Institutional Animal Ethics Committee (IAEC), L. M. College of Pharmacy, Ahmedabad, India, in compliance with the guidelines of the Committee for the Purpose of Control and Supervision of Experiments on Animals (CPCSEA), Ministry of Fisheries, Animal Husbandry and Dairying, Government of India, New Delhi. The study was performed on New Zealand rabbits (n = 3) in accordance with OECD guidelines, wherein sterile drug-loaded contact lenses and sterile placebo contact lenses were carefully applied to the right eye of each rabbit, while the left eye remained untreated to serve as a control. To evaluate the possible irritancy of the developed formulation, the animals were routinely checked for signs of ocular irritation, such as redness, swelling, and watering.

### 2.10. Ex Vivo Study

Fresh goat eyeballs preserved in cold, sterile normal saline and collected within two to three hours of enucleation were used in an ex vivo drug release study. Prior to experimentation, the eyes were rinsed with STF, and only intact corneas without damage were selected. The anterior segment, including the cornea with a scleral rim, was carefully excised and mounted between the donor and receptor compartments of a Franz diffusion cell, with the epithelial side facing the donor chamber. The receptor chamber was filled with STF, pre-warmed to 34–37 °C and continuously stirred using a magnetic stirrer (Remi Equipment Pvt. Ltd., Mumbai, India) to simulate physiological ocular conditions. Optimized EPL-loaded NLCs-laden contact lens formulations along with control batches were carefully placed on the corneal surface, and samples were withdrawn from the receptor chamber at predetermined intervals, with fresh STF immediately replaced to maintain sink conditions. The collected samples were analysed by UV–Vis spectrophotometry to quantify EPL release, while temperature and sterility were maintained throughout the study.

### 2.11. Cytocompatibility and Stability Test

The cellular biocompatibility of leached content from EPL-loaded NLCs-laden contact lens was tested using an indirect method against Arising Retinal Pigment Epithelia cells (ARPE-19) as reported previously [27]. In brief, sterile EPL-loaded NLCs-laden contact lens was transferred into 2500 μL of DEMF-12 complete cell culture media pre-added in a six-well culture plate and left for 24 h at 37 °C in CO_2_ incubator. The released content was used for the treatment of epithelia cells. The ARPE-19 cells were seeded at a density of 10^4^ in 96-well plate and incubated for 24 h at 37 °C in CO_2_ incubator. The monolayer confluence of the cells was treated with the content released from EPL-loaded NLCs-laden contact lens in two-fold serial dilutions, while intreated cells were tested as control for 24 h. The spent media was replaced with of 0.5 mg/mL MTT dissolved in incomplete DEMF-12 after 24 h of incubation at 37 °C in CO_2_ incubator. The media was further replaced with DMSO to initiate the conversion of formazan crystals by living cells in presence of DMSO. The OD was measured at 560 nm using spectrophotometer. Moreover, the stability of optimised EPL-loaded NLCs-laden contact lens was stored in STF at 4 °C for 30 days and tested for zeta potential, size, and EE (%).

### 2.12. Statistical Analysis

All experiments were performed in triplicate and presented as mean ± standard deviation.

## 3. Results and Discussion

### 3.1. Solubility Study of EPL in Solid Lipid, Liquid Lipid and Surfactant

**Figure 2:**
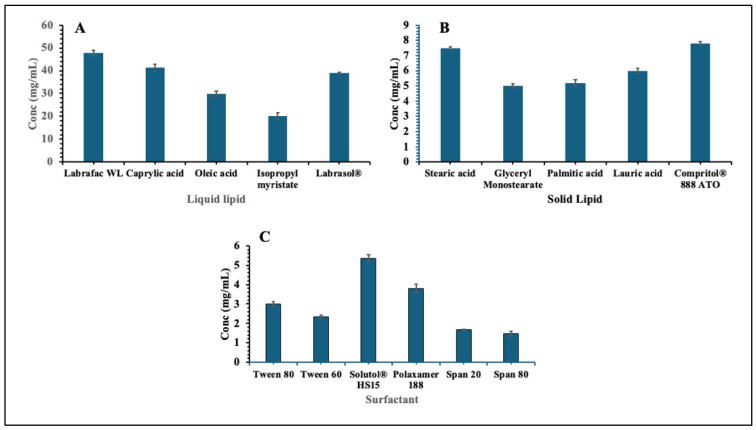
Solubility study of EPL in liquid lipid (**A**), solid lipid (**B**), and surfactant (**C**).

The solubility study of EPL was performed to identify suitable excipients for the development of NLCs. The evaluation was carried out following the standard procedure for the selection of solid lipids, liquid lipids, and surfactants. The solubility of EPL in different excipients was determined, and the results are summarized in Figure 2. Among the tested excipients, Labrafac WL1349 exhibited the highest solubility of EPL among the liquid lipids, while Compritol 888 ATO showed the highest solubility among the solid lipids. For tested surfactants, Solutol HS15 demonstrated superior solubilizing capacity, compared to the others tested. Based on these findings, Labrafac WL1349 (liquid lipid), Compritol 888 ATO (solid lipid), and Solutol HS15 (surfactant) were selected for the formulation of EPL-loaded NLCs.

### 3.2. Data Analysis of Response (Y1): Globule Size

The size of the NLC globules is a key factor that affects their stability, how well they are tolerated in the eye, and how the drug is released. Analysis using a reduced quadratic model showed that the model was highly significant, with an F-value of 17.86 (*p* < 0.0001). This means that the changes in globule size were mainly due to the formulation components rather than random experimental variations. Both the individual effects of the lipids and surfactants, as well as their interactions, were found to be significant, highlighting the complex way in which these ingredients influence globule size.

The model proved reliable based on the fit statistics. The R^2^ value of 0.7592 indicated a strong match between the predicted and experimental results, while the adjusted and predicted R^2^ values (0.7167 and 0.6512, respectively) were close enough to show the model could reliably predict outcomes. The adequate precision ratio of 11.375, much higher than the minimum requirement of 4, confirmed that the model had a strong signal and was suitable for exploring the design space. The regression equation for globule size (Y1) is:

Y1 = (106.31) X1 + (14.66) X2 + (67.92) X3 − (327.90) (X1) (X3)

**Figure 3:**
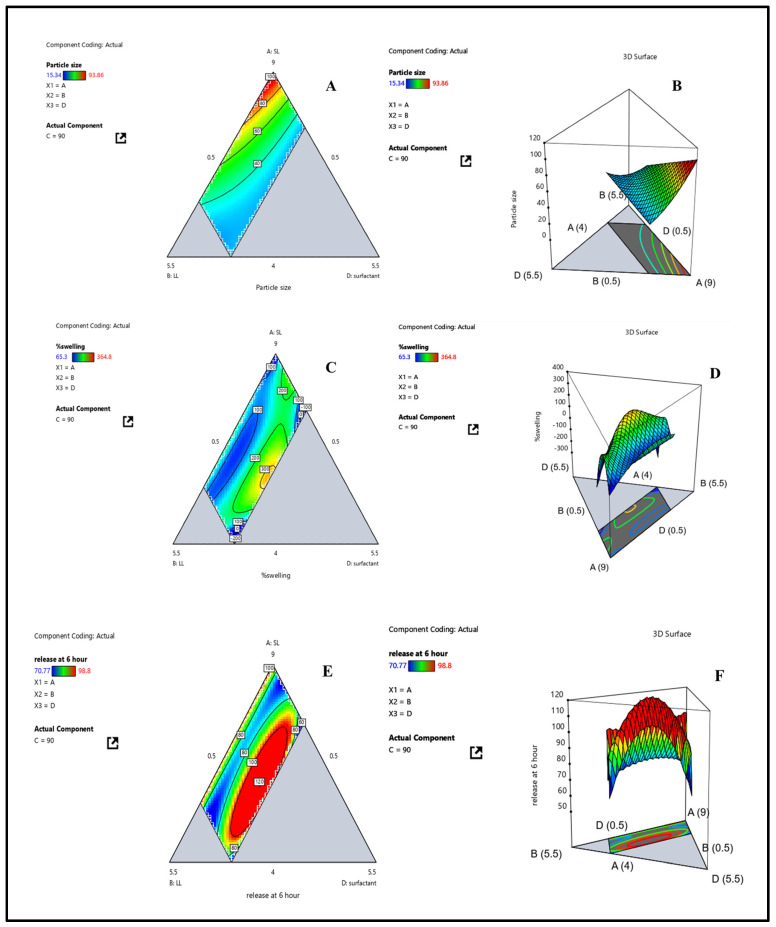
Contour plots representing the responses (**A**) globule size (Y_1_), (**C**) swelling index (Y_2_), and (**E**) drug release at 6 h (Y_3_); and 3D surface plots representing the responses (**B**) globule size (Y_1_), (**D**) swelling index (Y_2_), and (**F**) drug release at 6 h (Y_3_).

Regression coefficient analysis showed that solid lipid (X1) had the strongest effect, with larger globule sizes resulting from higher concentrations, most likely because of increased viscosity and decreased emulsification efficiency. On the other hand, surfactant (X3) promoted the development of more compact, stable droplets. Crucially, the negative interaction between surfactant and solid lipid suggested that surfactant could effectively offset the size-enlarging effect of solid lipid, encouraging the development of nanoscale droplets. Globule size varied experimentally between 15 and 94 nm for all formulations, indicating how sensitive this parameter is to changes in composition. The results support earlier studies that showed surfactant-rich systems produced smaller globules with better stability and optical clarity, which made them more appropriate for use in eyes [29]. The effects of formulation variables (X1, X2, X3) on globule size (Y1) were further demonstrated by contour and 3D surface plots (Figure 3A,B). The plots showed that globule size was considerably reduced by decreasing X1 and increasing X3, and that surfactant levels successfully counteracted the enlargement brought on by solid lipid. This emphasizes how crucial it is to maximize lipid–surfactant ratios in order to produce droplets that are between 30 and 40 nm in size. These droplets are ideal for improved stability, ocular tolerance, and effective drug delivery. Overall, the study found that surfactant functions as a stabilizing agent that modifies particle dimensions, while solid lipid concentration is the primary factor affecting globule size. Therefore, when designing NLC for ocular drug delivery, especially when integrated into contact lenses, the precise balance of lipid and surfactant concentrations is essential.

### 3.3. Data Analysis of Response (Y2): Swelling Index

A crucial feature of contact lenses is the swelling index, which has a direct impact on wearer comfort, hydration, oxygen permeability, and drug release capacity [30]. The swelling index model exhibited moderate predictive power, as reflected by value of R^2^ (0.7433), which may be attributed to experimental variability or block effects across formulation batches. The overall model with moderate predictive power remained statistically significant (F = 4.34, *p* = 0.0115) with a non-significant lack of fit (*p* = 0.6692), confirming its adequacy in explaining experimental observations. The adequate precision value of 8.93 further indicated a strong signal-to-noise ratio, ensuring the model’s reliability for design optimization. The wide experimental range of swelling index (65.3–364.8%) also contributed to data dispersion, reflecting the complex interplay between formulation variables. In particular, the strong influence of surfactant concentration and its interactions with lipid components created nonlinear behaviour that challenged model prediction but accurately captured the multifactorial nature of hydrogel swelling. Therefore, while the model’s predictive capability was somewhat constrained by high variability and interactive effects, it effectively described the key formulation trends governing the swelling behaviour of nanostructured lipid–laden contact lenses

The regression analysis yielded the following polynomial equation for swelling index (Y2):

Y2= (20.01) X1 + (−30.44) X2 + (−9711.13) X3 + (13,175.28) (X1) (X3) + (12,800.54) (X2) (X3) + 744.28 (X1) (X2) + (− 27,072.49) (X1^2^) (X2) (X3) + (− 21,697.95) (X1) (X2^2^) (X3) + (94,133.53) (X1) (X2) (X3^2^)

Swelling index values in experiments varied widely and were highly dependent on formulation composition, ranging from 65.3% to 364.8%. Surfactant concentration (X3) had the strongest effect among the variables, followed by liquid lipid (X2), while solid lipid (X1) had a relatively minor impact. Elevated solid lipid concentrations limited swelling because of increased matrix rigidity and decreased water penetration, whereas higher surfactant levels increased hydrophilicity, which in turn increased water uptake and swelling. Notably, the complexity of swelling behavior was emphasized by significant interaction terms. Surfactant could improve swelling by counteracting the constraining effects of lipids, as indicated by positive coefficients for X1 × X3 and X2 × X3 interactions. Large negative coefficients, on the other hand, for higher-order interactions (such as X1^2^X2X3 and X1X2^2^X3) indicated that too many lipid–surfactant combinations encouraged the formation of denser networks, which decreased free volume and made it more difficult to absorb water. These results were confirmed by contour and 3D surface plots (Figure 3C,D), which demonstrated that while swelling was decreased by higher solid lipid content (X1), swelling index rose with higher surfactant (X3) and liquid lipid (X2) concentrations. Higher-order interactions limited hydration by structurally densifying the hydrogel matrix, while the synergistic effects of X1 × X3 and X2 × X3 enhanced it. Overall, the analysis showed that lipid components influence swelling through both direct and indirect effects, while surfactant concentration is the primary factor governing swelling behavior. For contact lenses to have the desired swelling characteristics that guarantee hydration, transparency, and prolonged drug release, surfactant–lipid ratio optimization is therefore essential.

### 3.4. Data Analysis of Response (Y3) Drug Release at 6 h

A reduced quadratic model with solid lipid (X1), liquid lipid (X2), and surfactant (X3) as independent variables was used to assess drug release at 6 h (Y3). ANOVA showed that the model was statistically significant (F = 3.00, *p* = 0.0424), confirming that formulation factors, not experimental noise, were the main cause of variability in drug release. The model’s suitability was further confirmed by the non-significant lack-of-fit test (F = 1.16, *p* = 0.4147). The polynomial regression equation describing drug release at 6 h was as follows:

Y3 = (101.81) X1 + (107.93) X2 + (−9045.15) X3 + (15,862.11) (X1) (X3) + (16,185.67) (X2) (X3) + (−37.91) (X1) (X2) + (−14,519.41) (X1) (X2) (X3)

All three factors had a significant impact on drug release, according to the coefficient analysis, with surfactant concentration (X3) having the most pronounced effect. Increasing lipid concentrations encouraged release, presumably by improving drug solubilization within the lipid matrix, according to positive coefficients for X1 and X2. Conversely, the high negative coefficient for X3 indicated that increased surfactant levels by themselves decreased re-lease, most likely because of the drug’s micellar entrapment, which limited diffusion. Interaction terms were crucial. When paired with lipids, surfactant improved drug dispersion and partitioning within the matrix, facilitating drug release, as indicated by positive coefficients for X1 × X3 and X2 × X3. On the other hand, the negative three-way interaction term (X1 × X2 × X3) indicated that too many lipid surfactant combinations might limit diffusion by forming a denser microstructure or increasing viscosity, which would impede release. A sufficient signal-to-noise ratio was confirmed by R^2^ = 0.667 and adequate precision = 5.15, which both exceeded the 4 threshold and supported model reliability. Regression outputs and response surface plots matched experimental observations. Higher levels of solid (X1) and liquid lipid (X2) enhanced drug release, whereas excessive surfactant (X3) reduced it. Together with X1 and X2, X3 was crucial in modulating re-lease, as shown by the contour and 3D surface plots (Figure 3E,F). The necessity of carefully balancing lipid–surfactant ratios is highlighted by the observed antagonistic (X1 × X2 × X3) and synergistic (X1 × X3, X2 × X3) interactions. Overall, the study demonstrated that surfactant–lipid interactions are the primary determinant of drug release from NLCs-laden contact lenses at 6 h. These results are in line with earlier studies on drug delivery systems based on lipids, where surfactants control the kinetics of solubilization, partitioning, and release. A logical framework for accomplishing regulated and continuous ocular drug delivery is provided by optimizing these interactions.

### 3.5. Optimization of NLC-Laden Contact Lens Using D-Optimal Design

**Figure 4:**
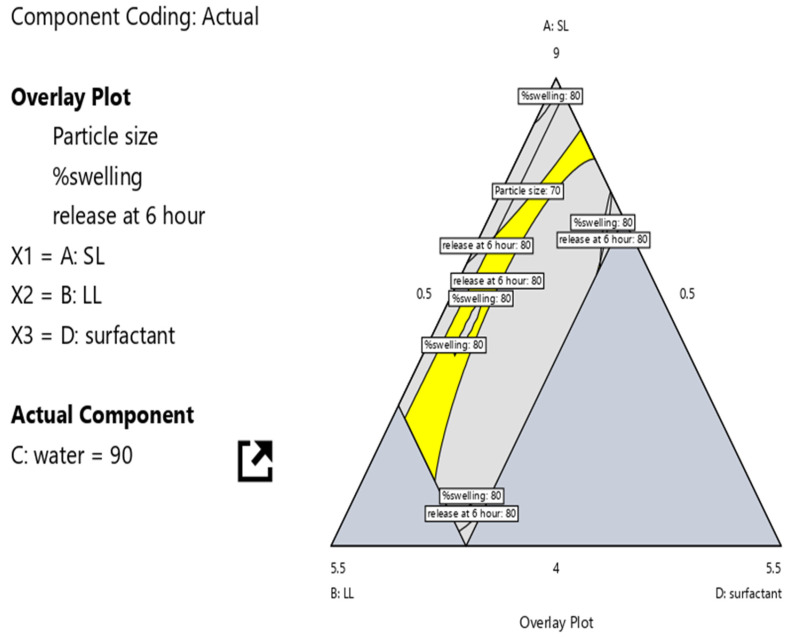
Overlay plot of the optimized batch.

Statistical analysis was performed to evaluate the effect of X1 (Compritol^®^ 888 ATO), X2 (Labrafac™ WL1349), and X3 (Solutol^®^ HS15) on Y1 (globule size), Y2 (equilibrium water content), and Y3 (drug release). Considering all constraints, the D-optimal mixture design identified the optimized formulation with a desirability. The optimized values were X1: 0.519 g, X2: 0.369 mL, and X3: 0.110 mL, which achieved Y1: 41.85 ± 2.14 nm, Y2: 140.69 ± 4.32%, and Y3: 79.23 ± 1.25%. The overlay plot for the optimization of NLCs-laden contact lenses is shown in Figure 4.

### 3.6. Characterization of Batches of EPL Loaded Nanostructured Lipid Carrier

#### 3.6.1. Globule Size and Polydispersity Index Analysis

**Figure 5:**
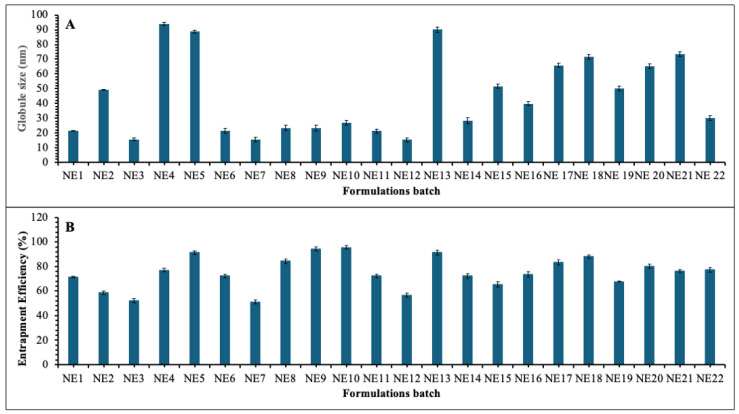
Characterization of EPL-loaded NLCs globule size distribution (**A**) and entrapment efficiency (**B**).

The globule size and polydispersity index (PDI) of the EPL -loaded nanostructured lipid carrier (NLC) formulations were measured using a Malvern Zetasizer Nano ZS-90. The mean globule size of the formulations varied between 15.34 nm (NE12) and 93.86 nm (NE4) as shown in Figure 5A, reflecting the influence of lipid-to-surfactant ratios on droplet characteristics. Formulations containing a moderate concentration of solid lipid (0.43–0.66 g), higher levels of liquid lipid (0.23–0.40 mL), and adequate surfactant concentrations (0.16–0.20 mL) consistently yielded nanometric droplets below 25 nm. To produce uniformly distributed nanosized droplets, representative batches like NE3 (15.53 nm), NE7 (15.40 nm), and NE12 (15.34 nm) showed effective emulsification and stabilization. While increasing surfactant concentration lowers interfacial tension between lipid and aqueous phases, resulting in smaller particles, higher liquid lipid concentrations reduce particle size by decreasing the viscosity of the internal phase [31]. The droplet sizes of formulations with reduced liquid lipid (<0.13 mL), excessive solid lipid (≥0.74 g), and insufficient surfactants (<0.10 mL) were significantly larger, as seen in NE4 (93.86 nm) and NE5 (88.69 nm). This increase was ascribed to droplet aggregation during emulsification caused by incomplete surfactant coverage of the lipid interface. The formulations’ PDI values, which varied from 0.311 to 0.563, confirmed primarily narrow size distributions. However, as seen in NE4 (93.86 nm) and NE5 (88.69 nm), formulations with reduced liquid lipid (<0.13 mL), excessive solid lipid (≥0.74 g), and insufficient surfactants (<0.10 mL) showed noticeably larger droplet sizes. This rise was ascribed to droplet aggregation during emulsification caused by incomplete surfactant coverage of the lipid interface. Primarily narrow size distributions were confirmed by the formulations’ PDI values, which varied from 0.311 to 0.563.

#### 3.6.2. Entrapment Efficiency

The EE% values (Figure 5B) differed significantly across the various batches, mainly because the solid lipid, liquid lipid, and surfactant compositions were different and affected the size of the globules. Formulations such as NE9 (94.3%), NE10 (95.6%), and NE5 (91.4%) demonstrated the highest entrapment efficiency. Their moderate surfactant levels (0.16–0.2 mL), lower liquid lipid content (≤0.08 mL), and higher solid lipid content (≥0.79 g) may be the cause of this. These parameters favored the development of a more compact and rigid lipid matrix, effectively entrapping the drug despite the presence of slightly larger globule sizes (23–26 nm). According to studies, formulations with a higher solid lipid content show noticeably better entrapment efficiency, and solid lipids form more densely and ordinately packed structures that successfully retain drug molecules. Since more regular and densely packed lipid structures exhibit superior drug retention compared to less ordered systems, the formation of compact, rigid lipid matrices at higher solid lipid concentrations is essential for drug retention [32]. Conversely, formulations like NE3 (52.3%) and NE7 (51.2%) exhibited significantly lower EE %. These batches had higher liquid lipid contents (0.31–0.4 mL), higher surfactant levels, and comparatively lower amounts of solid lipid (0.43–0.49 g). Despite encouraging smaller globule sizes (~15 nm), these conditions also resulted in less compact internal structures, which decreased entrapment capacity and allowed drug leakage. Research confirms that higher liquid lipid content disrupts the formation of pristine lipid crystals, creating pathways for drug migration to the external phase due to reduced viscosity and heightened molecular mobility [33]. The imperfect crystal structure created by excessive liquid lipid, while providing more loading space, can facilitate drug expulsion during storage and compromise structural integrity [34]. The results suggest that higher solid lipid concentrations and the formation of compact lipid matrices enhanced drug entrapment, whereas increased liquid lipid and excessive emulsification favor drug release but compromise entrapment efficiency. The entrapment efficiency of optimized batch was found to be 93.32 ± 1.27%.

### 3.7. Characterization of EPL Loaded Nanostructured Lipid Carrier Contact Lens

#### 3.7.1. Swelling Study

**Figure 6:**
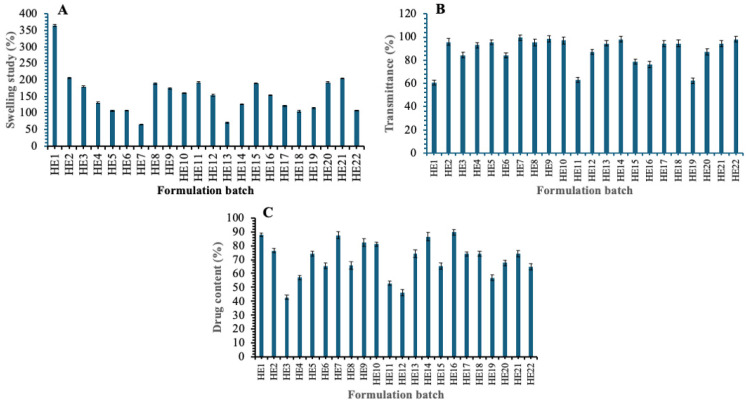
Characterization of EPL-loaded NLC contact lens swelling study (**A**), transmittance study (**B**), and drug content (**C**).

The swelling behaviour of EPL-loaded NLCs-laden contact lens (HE1–HE22) was evaluated to determine the effect of formulation variables on the hydration properties of the hydrogel matrix, and the results are shown in Figure 6A. The swelling values ranged from 65.3% (HE7) to 364.8% (HE1), indicating wide variability among the formulations. Formulations containing moderate levels of solid lipid (0.56–0.66 g) in combination with adequate liquid lipid (0.22–0.31 mL) and higher surfactant concentrations (0.16–0.20 mL) generally exhibited greater swelling, with values often exceeding 180% (e.g., HE8: 189.23%, HE11: 192.56%, HE15: 189.32%, and HE21: 204.2%). In contrast, contact lenses prepared with higher solid lipid content (>0.74 g) and lower surfactant levels (<0.10 mL), such as HE4 (131.1%) and HE5 (106.9%), showed reduced swelling, suggesting restricted water uptake due to tighter lipid packing within the hydrogel network. The lowest swelling values were observed in HE7 (65.3%) and HE13 (70.23%), irrespective of liquid lipid variation, indicating that surfactant concentration is a more decisive factor than liquid lipid content in governing hydrogel hydration. These results imply that the proportions of solid, liquid, and surfactant lipids are crucial in controlling the swelling capacity of NLCs loaded contact lenses, which in turn affects the lenses’ comfort, hydration, and possible drug release properties. Research demonstrates that the amount of surfactant greatly affects how hydrogels swell, with higher concentrations enhancing their hydrophilicity and capacity to absorb water [35]. Because of improved structural compactness and smaller pores, lipid nanoparticle-filled hydrogels become harder and absorb less water when their solid lipid content increases [36]. The optimised batch’s swelling index was 140.69 ± 4.32%.

#### 3.7.2. Transmittance Study

Percent transmittance at visible wavelength was used to assess the optical clarity of EPL-loaded NLCs-laden contact lens, the results are shown in Figure 6B. Depending on the lipid and surfactant composition, the transmittance percentage values varied significantly between formulations, ranging from 60.6% (HE1) to 99.6% (HE7). High transmittance values above 90% were shown by the majority of formulations, suggesting adequate transparency appropriate for ocular application. Representative batches such as HE2 (95.6%), HE5 (95.5%), HE7 (99.6%), HE9 (98.5%), HE14 (98.2%), and HE22 (98.2%) showed excellent optical clarity, comparable to conventional contact lenses. The reduced transparency of some formulations, such as HE1 (60.6%), HE11 (63.3%), and HE19 (62.4%), on the other hand, may have been caused by increased light scattering due to heterogeneous dispersion or higher solid lipid loading within the hydrogel matrix. Batches like HE3 (84.4%), HE6 (84.1%), HE12 (87.2%), and HE20 (87.2%) showed intermediate clarity, indicating a partial impact of lipid and surfactant balance on light transmission. The findings show that most formulations kept optical transmittance within the permissible range (>90%), indicating their potential applicability for drug delivery and vision correction without sacrificing contact lens transparency. The optimized batch’s transmittance was 80.54 ± 1.12%.

#### 3.7.3. Drug Content

The drug content of EPL -loaded NLCs-laden contact lenses (HE1–HE22) was evaluated as percent assay to assess uniformity and incorporation efficiency and the results are shown in Figure 6C. The assay results showed variation in drug loading across formulations, ranging from 42.9% (HE3) to 89.6% (HE16). Formulations with moderate solid lipid (0.56–0.66 g) and sufficient surfactant (0.11–0.20 mL) generally exhibited higher drug content, as seen in HE1 (87.8%), HE7 (87.4%), HE14 (86.5%), HE16 (89.6%), and HE9 (82.4%). In contrast, formulations with disproportionate lipid ratios or lower surfactant content (<0.10 mL) tended to have less drug incorporation; HE3 (42.9%), HE4 (57.1%), HE12 (46.2%), and HE19 (56.0%) are examples of these formulations. Intermediate assay values (60–75%) were observed in multiple batches, including HE5 (74.3%), HE6 (65.3%), HE8 (65.7%), HE15 (65.3%), and HE20 (67.5%), suggesting that drug loading is sensitive to the balance between solid lipid, liquid lipid, and surfactant concentration. The findings show that formulation composition has a major impact on drug entrapment efficiency and that consistent and repeatable drug content in NLCs-laden contact lenses requires the right lipid-to-surfactant ratios. Optimal surfactant concentration enhances drug loading capacity by maintaining appropriate interfacial properties and preventing drug expulsion during formulation preparation, while insufficient surfactant leads to poor emulsification and reduced drug incorporation [36]. Balanced solid lipid concentration with adequate liquid lipid and proper solid-to-liquid lipid ratios promotes higher drug loading and prevents phase separation, whereas improper lipid ratios compromise drug accommodation within the nanoparticle matrix [36]. The drug content of optimized batch was found to be 96.32 ± 0.84%.

#### 3.7.4. In Vitro Release

**Figure 7:**
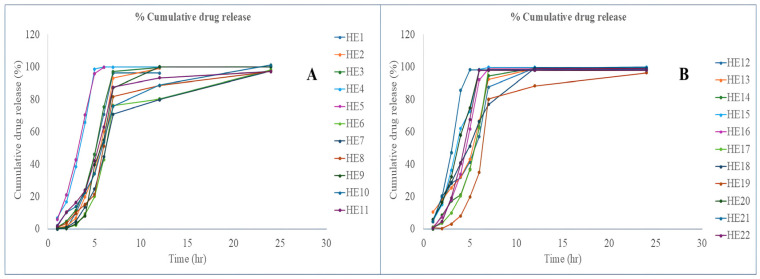
Drug release of EPL loaded NLCs-laden contact lens (HE1 to HE11) (**A**) and contact lens (HE12 to HE22) (**B**).

The cumulative drug release profiles (Figure 7) of EPL-loaded NLCs-laden contact lenses were evaluated. The release behavior varied according to the lipid–surfactant composition of the formulations. Formulations such as HE3, HE7, and HE12, which were prepared with smaller NLC globule sizes (~15.3–15.5 nm) due to moderate solid lipid content (0.43–0.49 g), higher liquid lipid levels (0.31–0.40 mL), and increased surfactant concentrations (0.17–0.20 mL), exhibited faster and more complete drug release. This trend corresponded with higher drug assay values in these lens batches. On the other hand, formulations that had larger globule sizes (65–94 nm) due to lower liquid lipid levels (≤0.13 mL), higher solid lipid content (≥0.74 g), and lower surfactant concentrations (0.05–0.13 mL) demonstrated slower and incomplete drug release, which was reflected in relatively lower assay values. These findings suggest that the lipid–surfactant composition, which controls NLC globule size, is a key factor in regulating drug diffusion from contact lenses. All things considered, the NLC system’s integration successfully managed the release rate, offering a long-lasting and consistent drug delivery profile appropriate for ocular application.

### 3.8. In Vivo Ocular Irritancy Test

**Figure 8:**
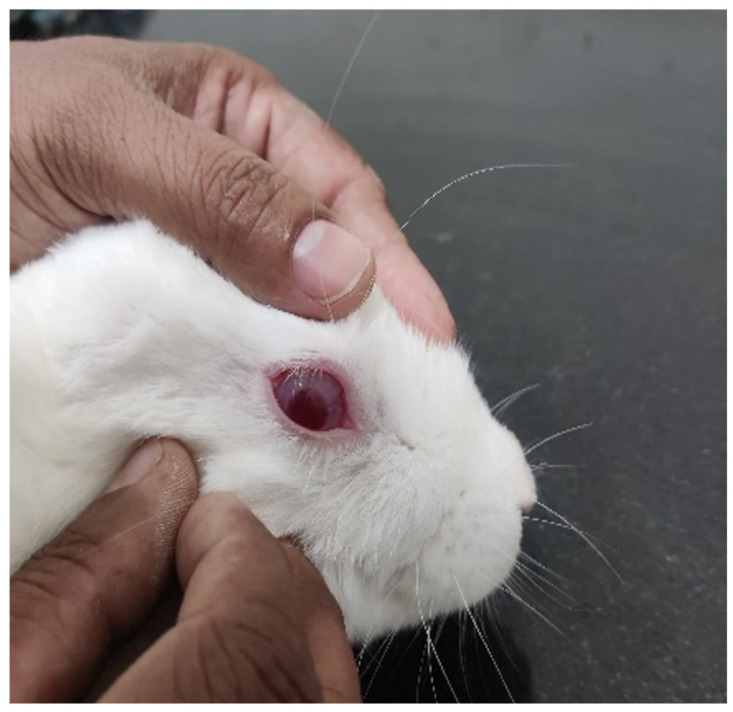
Contact lens instilled into rabbit’s cornea.

The ocular irritation potential of the NLCs-loaded contact lens formulation was evaluated in rabbits using the Draize eye irritation test in accordance with OECD guidelines. Each lens was carefully inserted into the conjunctival sac, as shown in Figure 8, and retained for 8 h, with visual observations recorded at 0.5, 1, 3, and 6 h. Both the NLCs-loaded and blank contact lenses showed no evidence of ocular irritation. No corneal opacity, iris damage, conjunctival redness, or chemosis was observed in any test eyes compared with controls. All tissues appeared normal, and irritation scores remained zero at all time points. These findings show that contact lenses, both NLCs-loaded and blank, are safe for use in the eyes and do not cause irritation.

### 3.9. Ex Vivo Permeability Study

To test EPL’s ex vivo corneal permeability, excised goat corneas were placed on a Franz diffusion apparatus. The control lenses exhibited a slow permeation profile, reaching only 38.14 ± 2.41% after 5 h. The optimised NLCs-loaded lenses, on the other hand, had a much higher permeation rate, reaching 75.73 ± 1.23% in 4 h and 97.26 ± 1.95% in 6 h. This enhanced permeability of NLCs-laden lenses can be attributed to the small size and high surface activity of nanostructured lipid carriers (NLCs), which increase drug solubility and facilitate easier drug passage across the cornea [37]. Drug partitioning into the lipophilic layers is facilitated by their ability to interact with corneal epithelial membranes due to their lipid nature [38]. Additionally, the prolonged release from the contact lens matrix decreases tear fluid washout by prolonging the drug’s stay on the corneal surface [39]. Additionally, surfactants have the ability to temporarily alter corneal tight junctions, greatly increasing penetration [40]. Together, these elements account for the significantly increased corneal penetration observed with NLCs-loaded lenses, demonstrating their potential to improve the ocular bioavailability of poorly soluble medications such as EPL.

### 3.10. Cytocompatibility and Stability Test

ARPE-19 cells were used to test the biocompatibility of the optimised EPL-loaded NLC-laden contact lens assay. The cells were more than 80% viable, according to the results, indicating good biocompatibility. These outcomes aligned with earlier research on EL-loaded oleogel [22]. Furthermore, the stability of the formulation was confirmed by the stability study results, which indicated negligible changes in size and zeta potential with an encapsulation efficacy of >80%.

### 3.11. Future Prospective

Future work will focus on further enhancing the therapeutic performance of EPL-loaded contact lenses by integrating advanced hydrogel materials such as chitosan or cyclodextrin, which offer mucoadhesive and antibacterial properties. These polymers may improve ocular retention, comfort, and microbial safety while enabling more controlled and prolonged drug release [41,42]. Additionally, in vivo pharmacokinetic and pharmacodynamic studies in diabetic retinopathy models will be pursued to validate long-term efficacy. Exploring stimuli-responsive lenses and scaling up manufacturing will also be key steps toward clinical translation, ultimately strengthening the potential of NLC-laden contact lenses as an effective platform for ocular drug delivery.

## 4. Conclusions

This study successfully illustrated the formulation and enhancement of EPL-loaded NLCs-embedded contact lenses for improved ocular drug delivery. This method allows for long-lasting and controlled drug release, better corneal permeation, and a high level of patient compliance. The optimized NLC system, which used Solutol HS15 as the surfactant and a mix of liquid and solid lipids, had good properties, such as nanosized globules, high entrapment efficiency, and a high drug content. The contact lenses showed better swelling and optical clarity, making them comfortable and clear for long periods of time. The formulation’s potential for controlled and efficient ocular delivery was confirmed by sustained EPL release over 24 h and improved corneal permeation. In vivo ocular irritation studies showed no signs of irritation, which supports the safety and biocompatibility of the system.

The NLC system’s optimization was validated by the D-optimal mixture design, highlighting its potential to enhance therapeutic outcomes in diabetic retinopathy. Future research could examine clinical translation, long-term efficacy, and application to additional lipophilic ophthalmic medications. Commercially speaking, the formulation and lens fabrication procedures are reliable, repeatable, and scalable, providing opportunities for industrial production. The developed NLCs-loaded contact lenses thus provide a promising platform for sustained, patient-friendly ocular therapy, aligning with the growing demand for advanced ophthalmic drug delivery systems.

## Figures and Tables

**Table 1:** Independent and dependent variables for the optimization of EPL-Loaded NLCs.

Independent Variable					
X1		X2		X3	
Compritol 888 ATO		LabrafacWL1349		Solutol HS15	
Low (g)	High (g)	Low (mL)	High (mL)	Low (mL)	High (mL)
0.43	0.87	0.05	0.4	0.05	0.2
Dependent variable					
Y1-Globule Size (nm)		Y2-Equilibrium Water content		Y3-Drug release at 6 h	

**Table 2:** Batches of nanostructured lipid carriers.

Batch	Solid Lipid (g)	Liquid Lipid (mL)	Surfactant (mL)	Water (mL)
NE1	0.58	0.22	0.2	9
NE2	0.64	0.31	0.05	9
NE3	0.49	0.31	0.2	9
NE4	0.77	0.18	0.05	9
NE5	0.87	0.08	0.05	9
NE6	0.66	0.23	0.11	9
NE7	0.43	0.4	0.17	9
NE8	0.67	0.13	0.2	9
NE9	0.79	0.05	0.16	9
NE10	0.79	0.05	0.16	9
NE11	0.66	0.23	0.11	9
NE12	0.43	0.4	0.17	9
NE13	0.87	0.08	0.05	9
NE14	0.66	0.23	0.11	9
NE15	0.56	0.31	0.13	9
NE16	0.56	0.39	0.05	9
NE17	0.74	0.13	0.13	9
NE18	0.74	0.13	0.13	9
NE19	0.5	0.39	0.11	9
NE20	0.71	0.24	0.05	9
NE21	0.74	0.13	0.13	9
NE22	0.61	0.27	0.12	9

## Data Availability

All experimental data is presented within the manuscript.
